# Glycosyltransferases and Transpeptidases/Penicillin-Binding Proteins: Valuable Targets for New Antibacterials

**DOI:** 10.3390/antibiotics5010012

**Published:** 2016-02-17

**Authors:** Eric Sauvage, Mohammed Terrak

**Affiliations:** Centre d’Ingénierie des Protéines, University of Liège, B6a, Quartier Agora, allée du six Août 11, 4000 Liège 1, Belgium; eric.sauvage@ulg.ac.be

**Keywords:** peptidoglycan, glycosyltransferase, transpeptidase, penicillin-binding proteins, β-lactam, lipid II, antibiotics resistance

## Abstract

Peptidoglycan (PG) is an essential macromolecular sacculus surrounding most bacteria. It is assembled by the glycosyltransferase (GT) and transpeptidase (TP) activities of multimodular penicillin-binding proteins (PBPs) within multiprotein complex machineries. Both activities are essential for the synthesis of a functional stress-bearing PG shell. Although good progress has been made in terms of the functional and structural understanding of GT, finding a clinically useful antibiotic against them has been challenging until now. In contrast, the TP/PBP module has been successfully targeted by β-lactam derivatives, but the extensive use of these antibiotics has selected resistant bacterial strains that employ a wide variety of mechanisms to escape the lethal action of these antibiotics. In addition to traditional β-lactams, other classes of molecules (non-β-lactams) that inhibit PBPs are now emerging, opening new perspectives for tackling the resistance problem while taking advantage of these valuable targets, for which a wealth of structural and functional knowledge has been accumulated. The overall evidence shows that PBPs are part of multiprotein machineries whose activities are modulated by cofactors. Perturbation of these systems could lead to lethal effects. Developing screening strategies to take advantage of these mechanisms could lead to new inhibitors of PG assembly. In this paper, we present a general background on the GTs and TPs/PBPs, a survey of recent issues of bacterial resistance and a review of recent works describing new inhibitors of these enzymes.

## 1. Introduction

**Figure 1:**
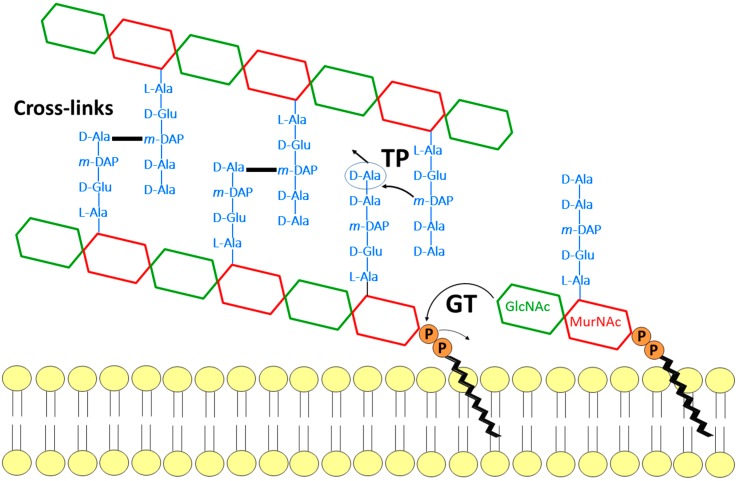
Schematic representation of the glycosyltransferase and transpeptidase reactions catalyzed by penicillin-binding proteins.

Peptidoglycan (PG) is an essential macromolecular sacculus surrounding most bacteria. PG determines the bacterium cell shape and provides protection from rupture under the high cytoplasmic osmotic pressure [1]. The PG structure consists of glycan strands made of alternating β-1,4-linked *N*-acetylglucosamine (GlcNAc) and *N*-acetylmuramic acid (MurNAc) residues cross-linked by peptides [1]. Nascent glycan strands are first polymerized by the glycosyltransferases (GTs) using the lipid II precursor (undecaprenyl-pyrophosphoryl-MurNAc-(pentapeptide)-GlcNAc) as a substrate [2,3], and cross-linked between them and with the pre-existing cell wall by the penicillin-binding proteins (PBPs)/transpeptidases (TPs) (Figure 1) [4,5]. The activities and regulation of GTs and TPs are coupled to provide a concerted knitting and enlargement of the PG sacculus [6,7,8,9,10].

Most of the steps in the PG biosynthesis pathway (from uridine diphosphate (UDP)-GlcNAc to the polymeric sacculus) are essential and some of them have been exploited as antibacterial targets [11], but the most successful of all and the most extensively targeted are the PBPs [4], named after the “miraculous” penicillin antibiotic that inactivates these enzymes. Since the discovery of penicillin and its wide utilization in medicine in the 1940s, several new classes and generations of related β-lactam antibiotics have been developed to successfully fight the emerging resistant strains. However, the widespread resistance to most β-lactams, particularly that mediated by β-lactamases, limits their efficacy unless they are used in combination with β-lactamase inhibitors, the most recent of which are mostly non-β-lactams [12,13]. Resistance affects all antibiotic classes, and the emergence of multidrug resistant bacteria (MDR) has become a major public health crisis worldwide [14]; therefore, the battle against MDR strains is a continuous endeavor that should explore all the present possibilities and develop new ones in order to identify new antibiotic classes with low resistance potential.

In addition to penicillin, several other antibiotics (glycopetides, lactibiotics, *etc.*) inhibit the last stage of peptidoglycan polymerization (GT and TP) by binding to the lipid II substrate, the last monomeric precursor of the peptidoglycan polymer, and preventing its use by the polymerases [15,16,17]. More recently, a newly discovered natural product, teixobactin, isolated from an uncultured soil microorganism (related to *Aquabacteria*), was found to inhibit cell wall synthesis by binding to the cell wall precursors lipid II and lipid III [18]. This mode of action will not be discussed further in this review and we refer the reader to recent reviews on the subject [15,17]. The examples cited here show that targeting PG synthesis by various modes of action is often used by microbes in the environment, and demonstrates once again that natural products are our best source of antibiotics [19]. Only a small portion of the microorganisms have been isolated, showing their huge potential as a natural product reservoir whose analysis through innovative techniques has just begun [18,20].

A deep knowledge of peptidoglycan metabolism, particularly networks decryption, a better characterization of all the potential targets involved, and the use of this knowledge to develop innovative screening assays, not only based on the inhibition of the enzymatic activity of the PBPs and other enzymes but also on the targeting of regulatory mechanisms, protein-protein interactions and the lipid II substrate, could lead to the new generation of antibacterial agents with novel mechanisms of action. PG synthases (GTs and TPs) are central to the complex networks of PG synthesis machineries (elongasome and divisome). Inhibition of the late stage of PG polymerization (GT-TP) has the advantage that the target is located outside the cytoplasmic membrane and therefore is more easily accessible to antibacterial agents, particularly in Gram-positive bacteria; the structures of PG and the enzymes involved in its synthesis are unique to bacteria and this lowers the incidence of unwanted side effects on eukaryotic cells. This review is focused on recent progress in the characterization and targeting of the GT and TP activities of PBPs via direct interaction with their active sites rather than their substrate and emphasizes their great potential for the future of antibiotic discovery.

## 2. Penicillin-Binding Proteins: Classification, Structure and Function

With the exception of bacteria lacking peptidoglycan (*Mollicutes, Dehalococcoides, Rickettsia*), which are also deprived of PBPs, and the particular case of *Chlamydiae*, which lack class A PBPs but contain a ring-like structure of peptidoglycan instead of the characteristic sacculus [21,22], all bacteria possess at least one PBP of each class and, in general, the PBP content correlates with bacterial morphology and cell cycle [23]. Gram-negative cocci (e.g., *Neisseria*) generally have only one PG-synthesizing machinery, called the divisome, which is active at the septum. Gram-negative rod-shaped bacteria have a second PG-synthesizing machinery active during the elongation phase, and thus possess at least two class A and two class B PBPs. More complex situations exist in Gram-positive bacteria where the various forms of life may require additional PBPs. In major pathogenic Gram-positive bacteria (*Staphylococcus aureus*, *Streptococcus pneumoniae* and Enterococci) the occurrence of acquired or endogenous PBPs, very weakly sensitive to β-lactam antibiotics while maintaining functional transpeptidase activity in the presence of the antibiotic *in vivo*, confer to the bacterium a high level of resistance [24]. The β-lactam-insensitive PBPs are only found in Firmicutes, mainly in *Bacilli* but also in some *Clostridia* (e.g., *C. difficile*). Antibiotherapy against clostridial infection is usually not based on β-lactams but infection with *C. difficile*, in which the existence of a β-lactam-insensitive PBP has not received the attention it deserves, and is induced by exposure to factors that disrupt gut microflora, including third generation cephalosporins [25].

Penicillin-binding proteins represent a vast group of proteins that are traditionally divided into high and low molecular weight (HMW, LMW) PBPs. Bipartition of PBPs is not simply a matter of protein size but rather reflects the different functions of these proteins: the late stages of peptidoglycan synthesis are achieved by HMW-PBPs while LMW-PBPs are mainly involved in the maintenance, remodeling and recycling of PG [4,26,27,28]. Generally, two HMW-PBPs (one class A and one class B) are necessary for the bacterium to survive, whereas deletion of all LMW-PBPs (class C), although deleterious, is not fatal to the bacterium [27]. As a result, HMW-PBPs are the primary targets of β-lactam antibiotics but LMW-PBPs may participate in the sensitization of the bacterium to these antibiotics [29,30] and simultaneous inhibition of HMW and LMW-PBPs may present an interesting synergistic effect.

**Figure 2:**
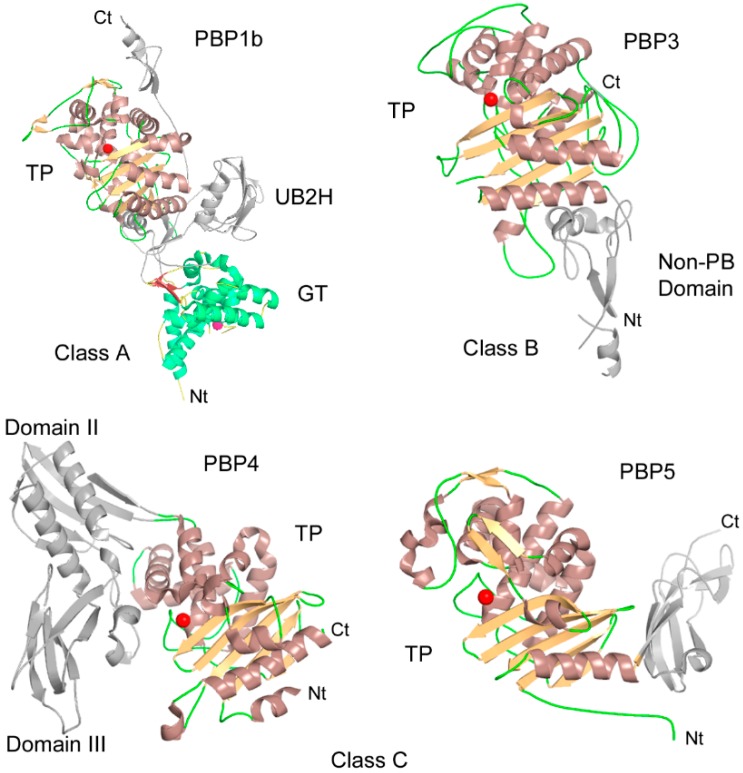
Structure of *E. coli* class A PBP1b [34], class B PBP3 [32], class C PBP4 [35], and class C PBP5 [36]. The TP domains are shown with brown helices and orange strands, the red sphere indicating the position of the active site serine. The PBP1b GT domain is shown with helices colored in lime green and strands in brick color. The GT catalytic centre (Glu233) is shown with a pink sphere.

PBPs are mostly multidomain enzymes divided in three classes (A, B and C) based on their domain organization and function (Figure 2). Subdivision of class B PBPs, based on their sequences, is of particular interest for understanding the problems of their inhibition and resistance. Subclass B1 (B1-PBPs) groups are the insensitive PBPs found in *Firmicutes* (e.g., *S. aureus* PBP2a). B2- and B3-PBPs are Gram-negative PBPs, respectively, active during elongation and division (e.g., *E. coli* PBP2 and PBP3). B4- and B5-PBPs are Gram-positive equivalents to B2- and B3-PBPs. In class A PBPs the transpeptidase domain is invariably linked to an N-terminal transglycosylase domain but additional domains can be encountered. The N-terminal module of class B PBPs has no enzymatic activity and was suggested to serve as a stalk, allowing the C-terminal TP domains to reach the peptidoglycan [31]. The N-terminal module is made of small domains and could be important for protein-protein interactions in the PG synthesis complexes [32]. The GT and TP activities are coupled within the same class A PBPs and also with their class B partners (e.g., PBP1a-PBP2, PBP1b-PBP3), showing regulated and synchronized PG synthesis [6,8,33].

In addition to the catalytic GT and TP domains, some PBPs, such as the class A PBP1a and PBP1b of *E. coli*, exhibit a small domain called UB2H (Figure 2) located between the GT and the TP domains [34]. They have been recently shown to function as the binding sites of regulatory lipoproteins LpoA and LpoB which stimulate the activities of PBP1a and PBP1b, respectively [37,38]. The Class B PBPs can also contain additional domains. B4-PBPS (e.g., PBP2x), for example, have two additional C-terminal PASTA domains (PBP And Serine/Threonine kinase Associated) [39]. These domains have been found to play a role in β-lactam binding, protein stability and bacterial morphogenesis [40,41,42].

The class C PBPs are found as single domains (type-7 PBPs; type AmpH) but an additional C- (type-5) or domains inserted in the PBP domain (type-4 PBPs) are also encountered. They have dd-carboxypeptidase and/or endopeptidase activity and are generally not essential for bacterial viability in laboratory conditions terminal domain but they play roles in the regulation of the degree of cross-linking in PG [4,28,43].

The overall structure of the PBP/TP domain consists of one alpha-beta and one all-alpha subdomain, with the active site located between both [4]. The transpeptidase activity relies on a few key residues of the three conserved motifs (SxxK, SxN, and KTGT) which decorate their active site [4,5]. The serine of the SxxK motif play an essential role in the two-step cross-linking reaction. Firstly, it performs a nucleophile attack on the CO of the terminal d-Ala-d-Ala amide bond of a pentapeptide which leads to the formation of an acylenzyme intermediate with departure of the terminal d-Ala; secondly, an amino group of a peptide from a neighboring glycan chain attacks this complex, resulting in the formation of the cross bridge and release of the free enzyme. Acylation of the active serine of PBPs by β-lactams blocks the enzyme in an inactive form for a long period of time. Modifications of the peptide side-chain of the peptidoglycan precursor, such as amidation or substitution, were found to be an important determinant of the specificity of the TP activity of some enzymes [44,45,46] and the efficacy of some antibiotics that interact with the substrate such as vancomycin [16,47].

The TP domain of HMW PBPs requires a peptide attached to the polymeric PG as a donor substrate for the cross-linking reaction, and they are not active on short peptides ending with d-Ala-d-Ala [3,48]. However, some LMW PBPs are able to catalyze transpeptidase activity using simple peptide substrates [49,50]. *S. aureus* PBP4 (LMW PBP) was shown to use lipid I, lipid II and PG strands as donors [48]. On the other hand, the TP reaction of almost all PBPs is less stringent for the acceptor substrate and can use various d-amino acids [49,51,52,53], allowing their incorporation (exchange) into PG, and in case of *S. aureus* PBP4 also into the lipid II and lipid II precursors. These properties have been exploited to label peptidoglycan and precursors by fluorescent d-amino acids in different organisms, providing a convenient tool to study peptidoglycan synthesis and bacterial morphogenesis in living cells [54,55].

## 3. Recent Developments in Transpeptidase Inhibitors

### 3.1. The β-Lactam Inhibitors of PBPs

**Figure 3:**
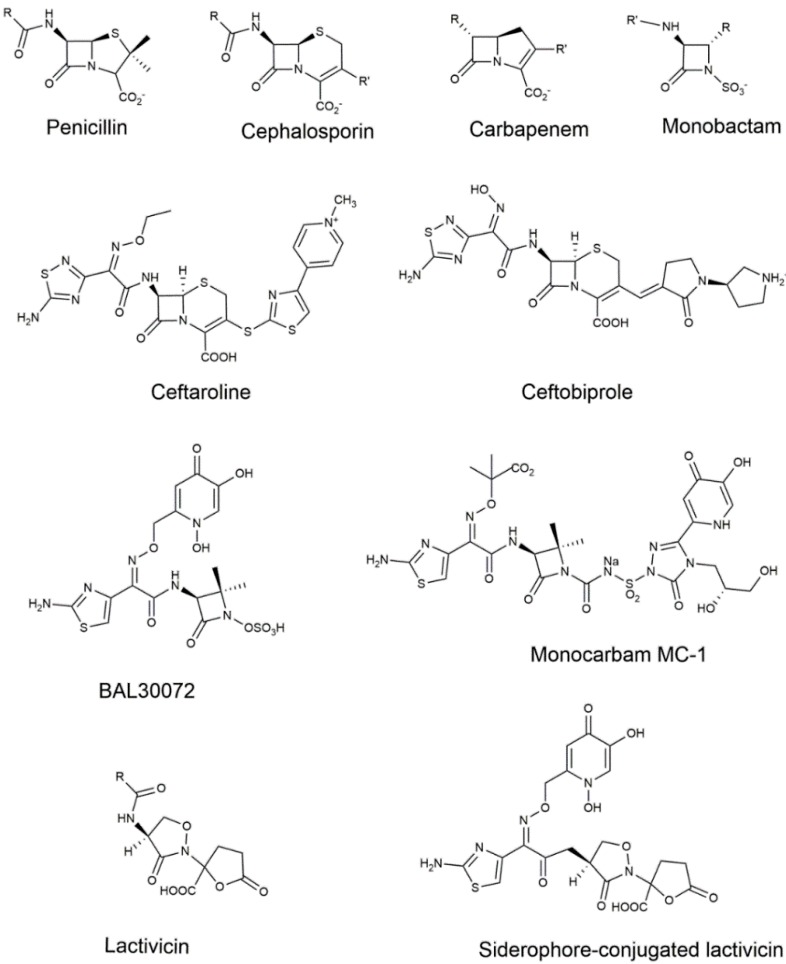
Structures of transpeptidase inhibitors.

Commercially available inhibitors of PBPs almost exclusively contain the four-membered β-lactam ring (Figure 3). Penicillins, cephalosporins, monobactams and carbapenems have been developed from natural representatives and, except for penicillin derivatives, research continues in an ongoing process to find new compounds active against the most harmful bacteria. The β-lactam antibiotics take advantage of their structural similarity to the d-Ala-d-Ala moiety of the natural substrate to lure the PBP [56].

#### 3.1.1. Cephalosporins

During the last decade, efforts in the development of new generation cephalosporins have culminated with the marketing of ceftaroline and ceftobiprole, two compounds active against methicillin-resistant *S. aureus* (MRSA). Ceftaroline has a broad-spectrum activity against Gram-positive pathogens and several Gram-negative pathogens, with the notable exceptions of *P. aeruginosa* and extended spectrum β-lactamase (ESBL)-producing Enterobacteriaceae [57,58]. Ceftobiprole is active against MRSA with broad spectrum activities against Gram-negative bacteria and other Gram-positive bacteria, in particular ceftriaxone-resistant streptococci [59].

Both compounds have been shown to bind to PBP2a [60,61] and to *E. faecium* PBP5 but with less affinity than to PBP2a [62,63]. In *S. pneumoniae*, ceftaroline binds to PBP2x [64], and ceftobiprole to all six PBPs, with a higher affinity for PBP2x [65]. The X-ray structure of PBP2a in complex with ceftobiprole [66] and with ceftaroline [67] provided the molecular details of the interaction between the compounds and the MRSA-acquired enzyme. Binding of both compounds involves a structural rearrangement of the active site residues, mainly a shift in the β3 strand and a twist of the residue backbone and side-chain atoms. The binding of ceftaroline in the active site of PBP2a possibly involves an allosteric site located in the non-penicillin-binding domain [68], but the existence of such an allosteric site was not reported upon binding of other β-lactams, either in PBP2a [69] or in PBP5fm [70].

Amino-acid mutations leading to resistance to ceftaroline in MRSA have rapidly emerged. In fact, moderately resistant strains predating the use of ceftobiprole have been reported [71]. The Glu_239_Lys mutation, located in the aforementioned allosteric site of the non-penicillin-binding domain, gives rise to a slight MIC increase (MIC > 2 mg/L whereas ceftaroline-susceptible MRSA have MIC ≤ 1 mg/L ); four strains with MIC > 8 mg/L were found with the additional Glu_447_Lys mutation, located in the penicillin-binding domain, close to the ceftaroline binding site [72]. Highly ceftaroline-resistant isolates (MIC > 32 mg/L) have been reported, bearing two mutations in the ceftaroline-binding pocket of the transpeptidase domain [73]. From a study conducted with 184 MRSA isolates, Lahiri *et al.* conclude that high level MICs are only achieved with mutations in the penicillin-binding domain whereas mutations in the non-penicillin-binding domain have only a small impact on ceftaroline susceptibility, probably via destabilization of a protein-protein interface [74].

The Glu_447_Lys mutation appears as crucial for reducing the sensitivity of PBP2a to ceftaroline. Interestingly, in *E. faecium* PBP5, a mutation in this region, consisting of the insertion of an additional serine after Ser_466_, leads to a highly insensitive PBP5 (MIC > 256 mg/L for ampicillin) [75]. Direct alteration of residues surrounding the active site of B1-PBPs is thus the most straightforward mean for staphylococci and enterococci to increase the level of resistance against the β-lactam antibiotics that threaten their survival.

Beyond ceftaroline and ceftobiprole, the last years have not seen the emergence of novel cephalosporins, even at the academic level. Nonetheless, there was a considerable rise in our understanding of the interaction between β-lactam antibiotics and Gram-negative PBPs of subclass B3 (Gram-negative PBP3 including *N. gonorrhoeae* and *N. meningitidis* PBP2), resulting from the solution of several structures in apo or complex form [32,76,77,78,79,80].

Han and colleagues have solved the structure of *P. aeruginosa* PBP3 with various compounds. They showed that the affinity of ceftazidime for B3-PBPs results from the fit of its aminothiazole ring into a pocket made of several aromatic residues. The residues lining the aminothiazole affinity pocket are conserved in B3-PBPs and, thus, compounds bearing a side-chain with an aminothiazole ring have a good chance to be active against Gram-negative bacteria. The ceftazidime side-chain has a carboxypropyloxyimino moiety but the 2-amino-4-thiazolyl methoxyimino (ATMO) side-chain is common to many third-generation cephalosporins. It resists the deleterious effect of class C β-lactamases and can be used for development of new β-lactamase inhibitors [81]. Unfortunately, the structure of a B3-PBP in complex with an ATMO side-chain-bearing compound (cefotaxime, ceftriaxone, …) has not been solved and one does not know if the methoxyimino moiety could fit into the aminothiazole ring binding pocket.

The class B PBP involved in cell division (FtsI) is the relevant target of cephalosporins and resistance due to alterations of FtsI is found in many non-β-lactamase-producing Gram-negative pathogens. Deletion of the *ftsI* gene has even been seen in rare cases of resistance in *Burkholderia pseudomallei* [82]. Mutations leading to resistant strains commonly occur close to the active site and, somewhat unexpectedly, mutations are specific to each bacterial species, *i.e.*, they do not overlap if the B3-PBPs structures of different species are superimposed. They are generally located in one of the two walls that border the active site entrance. On one side, the wall is made of the loop that acquires a stable conformation upon antibiotic binding (as discussed above), which covers β3 and β4 strands. Mutations conferring high β-lactam resistance are located in this loop (e.g., Glu545Ser in *N. gonorrhoeae*) or in contact with it (e.g., Arg517His and Asn526Lys in *H. Influenzae*). On the opposite side of the active site entrance, a long loop inserted between α4 and α5 helices covers the SxN motif. Reduced susceptibility to cephalosporins was recently reported in *E. coli* isolates as a result of the insertion of four residues in this loop [83]. As well, the Asp345a insertion in the equivalent loop of *N. gonorrhoeae* is commonly observed in penicillin-resistant strains, reducing the penicillin acylation rate approximately six-fold [84]. Interestingly, this insertion is located in the same region as the Ser466 insertion in PBP5 of highly resistant *E. faecium* strain.

Secondary mutations located in the penicillin-binding domain and sometimes in the non-penicillin-binding domain can reinforce the strain resistance. The subtle alterations that these mutations induce on the binding of antibiotics are not easy to explain. In transformable bacteria, especially *Neisseria* and Streptococci, target-mediated antibiotic resistance is exacerbated by the horizontal genetic exchange of fragments of chromosomal genes [85].

#### 3.1.2. Carbapenems

Carbapenems are last resort β-lactam antibiotics. Their broad spectrum activity and their high potency as antimicrobial agents arise from their ability to bind B2-PBPs as well as B3-PBPs [86,87]. Of note is their ability to also inhibit ld-transpeptidases [88,89,90]. ld-transpeptidases catalyze the formation of a 3,3 peptidoglycan cross-link instead of the classical 4,3 cross-link formed by dd-transpeptidases [91,92]. The 3,3 cross-link is predominant in the peptidoglycan of *M. tuberculosis* [93] and, in combination with clavulanic acid, carbapenems are bactericidal against drug resistant *M. tuberculosis*.

Unfortunately, the latest news makes the future rather bleak. With the spread of resistance mediated by β-lactamase production and emergence of new mechanisms of resistance, bacteria are winning the battle against the developers’ ingenuity. Resistance affects all carbapenems and perspectives of new effective carbapenems are scarce. New derivatives, awaiting further experimental evidence, have been proposed as the result of *in silico* analysis [94].

Resistance to carbapenems is essentially mediated by the production of β-lactamases. Carbapenemases were first found as metallo β-lactamases but all classes of β-lactamases are now found to contain carbapenemases [13]. Until the last months, a carbapenem-resistance mechanism resulting from PBP2 mutations had only been seen in an experimentally mutS- and tolC-deficient *E. coli* strain [95]. A recent paper reports a mutation in PBP2 as a new mechanism to render clinical isolates of *E. coli* non-susceptible to imipenem [96]. In PBP2, the substitution of the second motif alanine by a serine probably disrupts the interaction between the imipenem hydroxyethyl group and the aspartic acid present in motif 2 of B2-PBPs, reducing the affinity of imipenem for PBP2.

#### 3.1.3. Monocyclic β-Lactams

Although many monobactams have been developed, the only FDA-approved monobactam is aztreonam, which shows high antibacterial activity against Gram-negative bacteria but not against their Gram-positive counterparts. Aztreonam binds to Gram-negative B3-PBPs and the solution of the structure of the aztreonam: *P. aeruginosa* PBP3 adduct shows that the enzyme pocket that tightly accommodates the aminothiazole ring of the ceftazidime side-chain accepts the aminothiazole ring of aztreonam in the same way.

The latest efforts in the design of new monocyclic β-lactams have involved the incorporation of a siderophore group that exploits the iron-uptake systems to diffuse through the Gram-negative outer membrane and avoid resistance induced by efflux systems and low permeability mutations. The monosulfactam BAL30072 is a monobactam with a siderophore group connected to the methoxyimino moiety of the ATMO side-chain (Figure 3) [97]. It is active against *P. aeruginosa, A. baumannii* and KPC-possessing *Klebsiella pneumoniae*, with an enhanced activity when combined with meropenem or colistin [98,99,100]. BAL30072 has a high affinity for PBP3 but also for PBP1a and PBP1b [97]. Its aminothiazole group is expected to fit into the PBP3 active site, resulting in a high affinity for PBP3.

Along the same line, new monocarbams–monocarbams are monocyclic β-lactams with a carbonylaminosulfonyl activating group at the N-1 position (Figure 3) have been developed by Pfizer ([101], and references therein). MC-1 is a siderophore-conjugated monocarbam highly active against *P. aeruginosa* clinical isolates and the crystal structure of the adduct with PBP3 provided the structural basis for lead optimization [77]. Structure activity relationships have been explored for the triazolone-based siderophore-conjugated monocarbam, providing potent inhibitors of *P. aeruginosa* PBP3 with good pharmacokinetic properties [102]. Notwithstanding the valuable properties of siderophore-conjugated monocarbams, resistance could rapidly appear through mutations in the genes linked to various components of the siderophore-mediated iron uptake systems [103].

### 3.2. Non-β-Lactams

Non-β-lactam inhibitors of PBPs (reviewed in [104]) escape the action of β-lactamases but none have yet undergone developments sufficient for initiating clinical trials. The most promising non-β-lactam inhibitors of PBPs are certainly lactivicin analogs (Figure 3). Known for more than 30 years, lactivicin derivatives have exhibited antimicrobial activity against a wide spectrum of infectious bacteria [105]. Several derivatives resist the action of β-lactamases and may even behave as inhibitors of the latter [106]. Lactivicin analogs form a stable covalent adduct with the active site serine and crystal structures of adducts with *S. pneumoniae* PBP1b [107], and *P. aeruginosa* PBP3 and PBP1a [108] revealed the opening of both monocyclic cycloserine and γ-lactone rings upon acylation of the active serine. Recent developments have involved the incorporation of a siderophore moiety to benefit from the siderophore receptor uptake strategy (see chapter on monobactams) [108]. Incorporation of a hydroxypyridone moiety linked to the side-chain oxyimino group improved MICs in *P. aeruginosa* and *A. baumannii* strains. In addition to the opening of both rings as observed in PBP-lactivicin structures, the crystal structure with *P. aeruginosa* PBP3 showed a positioning of the aminothiazole identical to that observed with ceftazidime and aztreonam, whereas the ethylene carboxylate moiety resulting from the opening of the lactone ring and the dihydroxypyridone sideromimic moiety did not form strong interactions with the enzyme, allowing for further improvement of the compound. Interestingly, linking a presumably more efficient sideromimic (dihydroxyphthalimide instead of dihydroxypyridone) to the α-lactone position resulted in lower MICs, with inhibition potency extended to class A PBPs.

Other reactive centers have been shown to react with the active serine of PBPs. A boronic acid and a trifluoroketone bearing an appropriate peptidoglycan mimetic side-chain could form covalent adducts with the *Actinomadura* R39 dd-peptidase [109,110]. Other boronic acids were also designed and shown to bind to *S. pneumoniae* PBP1b [111]. Strikingly, the peptidoglycan mimetic boronic acid could not bind to HMW-PBPs [112], demonstrating the poor affinity of HMW-PBPs for their natural substrate; however, a ceftazidime or cefotaxime such as boronic acid might possibly do so. A ceftazidime such as boronic acid was shown to tightly bind to CTX-M-9 and CTX-M-16 class A β-lactamases [113], but, to our knowledge, it was not tested as an inhibitor of B3-PBPs.

Non-covalent inhibitors remain at a prospective level. Recent research, generally based on *in silico* screening, disclosed interesting compounds primarily targeting MRSA PBP2a. For example, the oxadiazoles were discovered by an *in silico* screening of compounds from the ZINC database [114], showing attractive properties against Gram-positive bacterial strains [115,116]. Macrocycle-embedded β-lactams also show moderate PBP inhibition properties with *S. aureus* PBP2a and *E. faecium* PBP5 [117].

PBP2a appears to use an allosteric site to trigger the active site opening [67,68]. This observation has stimulated the search for new compounds binding at the allosteric site with the help of molecular docking techniques [118,119]. The mechanism of action of these compounds and their potential to lead to clinically useful bactericidal drugs need further analysis.

In addition to *in silico* methods, a high-throughput screening assay was recently developed, allowing the screening of 50,000 compounds against *N. gonorrhoeae* PBP2 and the identification of seven non-β-lactam compounds active on penicillin-resistant gonococcal strains [120]. The assay is based on the interaction of a fluorescent β-lactam, Bocillin-FL, with solubilized PBPs, which results in an increase of fluorescence anisotropy in a time-dependent manner [121]. The assay can be monitored continuously in microtiter plates allowing the development of relatively high-throughput screening of TP inhibitors.

### 3.3. Synergistic Combinations

To circumvent the resistance to last resort antibiotics, synergistic use of β-lactams in combination with other β-lactams, β-lactamase inhibitors or other metabolic pathway–targeting drugs has been revived. These combinations can be beneficial not only in fighting resistant pathogens but also bacteria that are apparently susceptible *in vitro*, in particular clinical situations [122].

New inhibitors of β-lactamases and new inhibitors of PBPs have been combined, leading to original combinations that are targeted primarily against carbapenemase-producing Gram-negative pathogens. Several combinations are currently in clinical phase and two of them (ceftolozane/tazobactam and ceftazidime/avibactam) were recently approved by the FDA. We refer the reader to Bush’s recent review [12] for further information. Also covered by an extensive review, dual β-lactam therapy for Gram-negative bacteria is at least as effective as combinations of a β-lactam and an aminoglycoside, which is recommended for treatment of many serious Gram-negative infections [123]. Dual β-lactam therapy is not limited to Gram-negative bacteria but can also contribute to combating resistance in MRSA [124,125].

Novel small molecules were recently shown to potentiate β-lactam activity against various pathogens: β-cyclodextrin (β-CD) derivatives [126], fosfomycin [127], a molecule with a hypothetical cell wall synthesis–targeting function [128] and a positive interaction between inhibitors of protein synthesis and cefepime against MRSA [129].

## 4. Glycosyltrasferases Structure and Function

**Figure 4:**
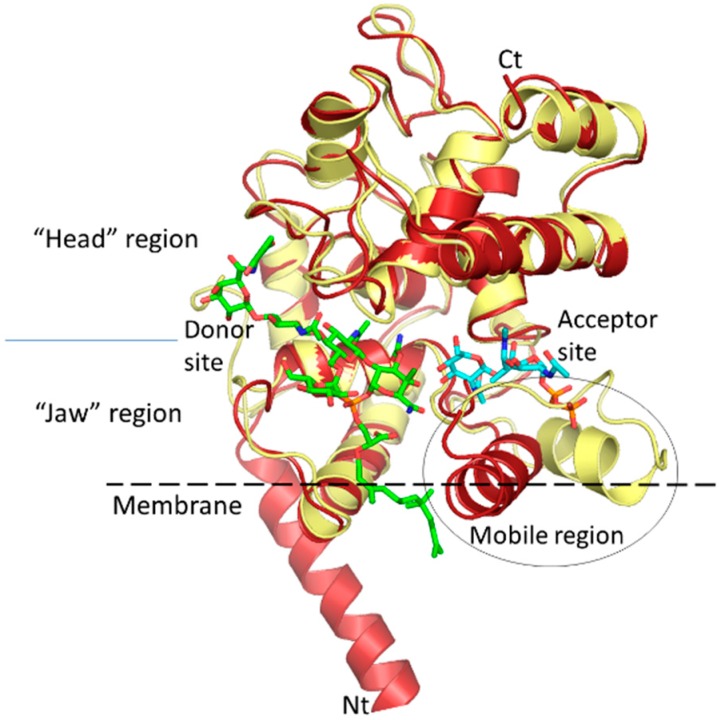
Superimposition of two structures of *S. aureus* MtgA, PDB 3VMR (yellow) and PDB 3HZS (red). Moenomycin (green) bound to the donor site and a lipid II analog (cyan) bound to the acceptor site are represented in stick. The mobile region between the two sites is highlighted by a circle.

The GT module is found in bacteria as a single domain called monofunctional glycosyltransferase or as the N-terminal domain of the multimodular class A PBPs. It contains about 200 amino acid residues folded into a structure much like that of the bacteriophage lambda lysozyme but, in contrast to the hydrolytic enzyme, it is endowed with a fascinating, highly processive polymerase activity converting the lipid II substrate to long glycan chains. Based on sequence similarities, these enzymes form family 51 in the GT classification database (Cazy database). Several crystal structures of bifunctional (GT-TP) PBPs and monofunctional GTs are now available, both in complex with the inhibitor moenomycin, lipid II analog or without a ligand [34,130,131,132,133]. The general structure is conserved among proteins from various species of microorganisms. The overall structure is composed mostly of alpha helices forming a globular domain with a large enzymatic cavity that divides this domain into two sub-domains (Figure 4). The features of the large one (“head” region) are reminiscent of those of the bacteriophage lambda lysozyme and the smaller one (“jaw” region) is characteristic of the family 51 GTs and contains a hydrophobic surface, suggesting that it is partially buried in the cytoplasmic membrane. Sequence alignment of GTs highlights five highly conserved consensus motifs [5]. Most of them are directed toward the enzymatic cavity and were confirmed to be important for interactions with substrate and moenomycin and enzymatic catalysis [3,132,133,134,135]. Six sugar units could be fitted into the GT enzymatic cavity based on the structure of the complex with moenomycin [136], which binds to the donor site. Of these, four sugar units belong to the donor substrate (the elongating glycan chain whose remaining chain extends beyond the GT domain and could reach the TP active site in bifunctional PBPs about 70 Å away) and two sugar units to the acceptor substrate, lipid II. The corresponding sub-sites are called donor and acceptor sites, respectively (Figure 4).

The catalytic mechanism of the polymerization reaction consists of a nucleophilic attack by the activated 4-OH group of the lipid II onto the C-1 carbon of the undecaprenyl pyrophosphate-linked glycan chain moiety (GlcNAc-MurNAc-peptide)_n_ (Figure 1), leading to the formation of a new β-1,4 glycosidic bond. This process is repeated after translation of the chain containing n + 1 disaccharide units which allow the binding of a new lipid II into the acceptor site (see below). Analysis of moenomycin binding in the presence of substrate analogs, mimicking the donor and acceptor substrates respectively, shows a cooperative mechanism between the two corresponding sites [137]. It has been also observed that preincubation of PBP1b with lipid IV (a donor which results from the condensation of two lipid II molecules) stimulates the initiation step of the GT reaction [138]. This suggests that binding of the donor substrate induces structural rearrangements, including probably in the acceptor site that facilitates the binding of lipid II during the elongation phase of processive polymerization. If the coupling reaction leading to the formation of the β-1,4 glyosidic bond is well established, the mechanisms of the translocation of the elongating glycan chain from the acceptor site to the donor site and of chain termination are less well understood. Recently, a newly identified *E. coli* lytic transglycosylase, MltG, was shown to interact with PBP1b and was proposed to function in glycan chain termination [139]. Also, it is not known how, *in vitro*, different GTs produce different glycan chain lengths characteristic of each enzyme [140,141]. This may be the result of different affinities of the enzyme for the elongating chain, with longer chains resulting from higher binding affinity.

GT crystal structures show a protruding and dynamic region between the donor and acceptor sites. The processive elongation of the glycan chain by the GT imposes several rounds of disaccharide-peptide additions before its release. This suggests that the intermediate products must shuttle from the acceptor site to the donor site after each reaction to allow the former to accommodate a new disaccharide. The dynamic protruding region could facilitate the translation of the product. On the other hand, the mobility of this region might be decreased by the binding of substrates in the active site [137].

## 5. Synthesis of Lipid II and Analogous Substrates, and Their Use to Study the GT

Lipid II is the immediate substrate of the GT, and its polymerization supplies the TP substrate. It must be available for the *in vitro* study of the GT and TP. Complete synthesis has been achieved [142,143,144,145,146,147,148]. Although the preparation of the lipid II substrate remains a tedious task, it has become more easily accessible for enzymatic and semi-enzymatic synthesis [145,149,150,151] because the limiting lipid moiety can be obtained in sufficient quantity from plant leaves [152]. In addition, MraY can use lipids of various sizes and structures, allowing variation and better solubility of the substrate [2,145,150,151,153,154]. These substrates can be labeled with radioactive isotopes in the peptide or the GlcNAc moieties, with various fluorescent chromophores attached to the peptide or with a dansyl group attached to the C20 polyprenyl [154]. These labeled substrates facilitated the development of activity assays [3,143,155,156,157]. The availability of substrate variants that serve as donor only, acceptor only, or that are not processed by the GT enzyme and thus function as inhibitors (see below) contributed greatly to the understanding of the specificity of the GT and TP catalyzed reactions [138,147,158,159,160,161,162,163]. Thanks to these substrates, lasting problems have been solved. For example, the elongation of the glycan chain by the GT at the reducing end of the growing chain was demonstrated experimentally [163,164]. It has also been shown that the donor and acceptor sites of the GT exhibit different requirements for the length of the lipid, with a minimum of 20 carbon atoms for the donor and a shorter size for the acceptor [153]. A lipid length of more than 20 carbon atoms was particularly critical for the GT processivity [153], and a C35 lipid was an even better substrate than a C55 one [145]. Pentaprenyl (C25, 2-cis and 2-trans) was not a substrate for GT but, when a benzene extension or a tetraprenyl one with a dansyl group extension at the end of the lipid chain, both considered C27 mimics, was added, , these analogs were GT substrates [154]. A penta-isoprene substrate with one isoprene in *trans* configuration was a better substrate than a penta-isoprene in all-*cis* configuration; this compound had a higher affinity towards several tested GT enzymes [156]. Analogs with two to four isoprene in *cis* configuration were poor substrates for most tested GTs [154]. Synthesis and evaluation of analogs of lipid II exhibiting different peptide sequences revealed that the d-lactate-l-Ala moiety of the peptide is required for the lipid II substrate to bind and be processed by the GT [165]. The synthesis of lipid IV labeled with ^14^C or a fluorophore [146,147,148] revealed important differences between *E. coli* PBP1a and PBP1b: while PBP1b uses lipid IV only in the presence of lipid II, PBP1a is able to couple two lipid IV molecules in the absence of lipid II [146].

## 6. Inhibitors of the Glycosyltrasferase

**Figure 5:**
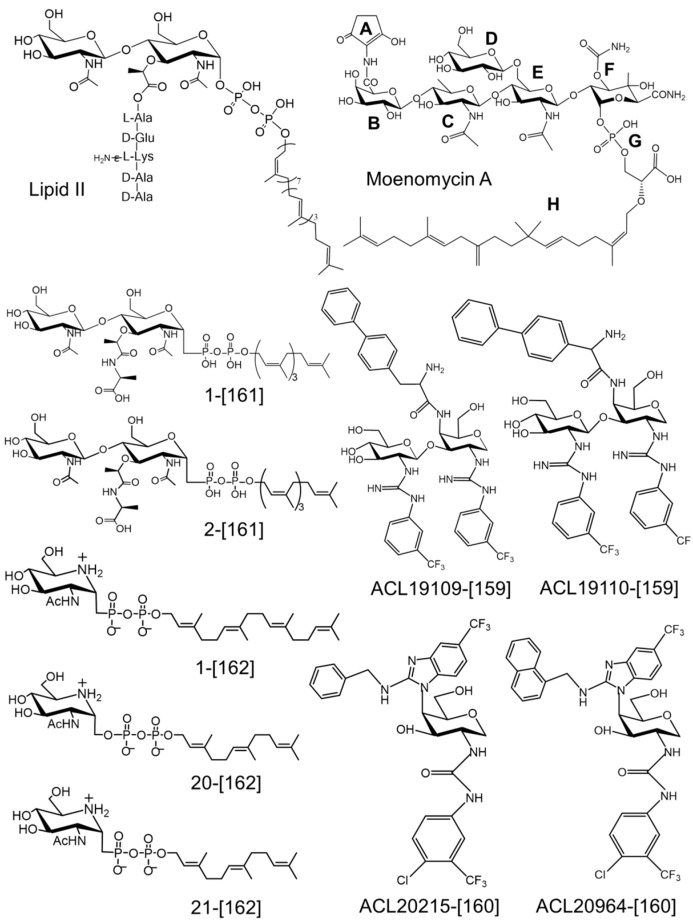
Structures of the lipid II substrate and the glycosyltransferase inhibitor moenomycin A and their recent synthetic analogs shown to inhibit GT activity. The compounds are numbered as in the original papers and the references are indicated in square brackets.

The GT51 is considered a validated target based on the essentiality of the class A PBPs in peptidoglycan synthesis in most bacteria (*E. coli, S. aureus, S. pneumoniae, etc.*…) and the antibacterial activity of moenomycin which binds specifically to the active site of the GT. Although in laboratory conditions the class A PBPs were not absolutely essential for the viability of *Bacillus subtilis* and *Enterococcus faecalis*—the presence of a novel GT was proposed [166,167]—they may be essential in the environment. Moenomycin did not pass the development trials for use in humans, and neither did any other GT inhibitor. Given the importance of the GT as a validated target, moenomycin was investigated in great detail, allowing a better understanding of the structure-activity relationships and inspiring the synthesis of new derivatives. The moenomycin A structure consists of a pentasaccharide (rings BCDEF) and a chromophore (ring A) linked to the moenocinyl lipid (H) via a phosphoglycerate (G) group (Figure 5). Its structure mimics the undecaprenyl-linked growing chain of the PG [168] and binds to the donor site of the GT enzyme [130] (Figure 4). The minimal structure that retains antibacterial and GT inhibition activities is the trisaccharide CEF-phosphoglycerate-lipid. Removal of ring C leads to a loss of antibacterial activity but retains significant activity *in vitro* [168]. The crystal structures of GT-moenomycin complexes show that most interactions are mediated by this trisaccharide fragment [130,136,169]. The two negatively charged groups of the phosphoglycerate moiety make critical contacts with conserved residues in the GT active site. These interactions were suggested to help orient the lipid chain for binding along the hydrophobic groove facing the cytoplasmic membrane. The lipid chain plays an important role in binding and its removal abolishes both antibacterial and *in vitro* GT activity [168].

Moenomycin is a highly potent inhibitor of the growth of several Gram-positive bacteria (MIC values generally below 1 µg/mL) [170,171]. However, some *Enterococcus faecium* strains are naturally resistant to moenomiycin [172]. Gram-negative bacteria are much less sensitive to moenomycin due to the presence of the outer membrane. Several GT from different microorganisms have been purified and tested *in vitro*, and most of them are inhibited by moenomycin at a nanomolar concentration ([3,156,173]. One exception is the non-essential MtgA of *E. coli* which does not appear to be inhibited by moenomycin [174].

Moenomycin was used as additive in feed stocks without major incidence on resistance development [175], suggesting that its mechanism of action is less prone to resistance. In laboratory experiments, moenomycin-selected resistant *S. aureus* mutants have point mutations in the GT domain of the essential PBP2 (Y196D being the most frequent mutation) [176]. This mutation causes a cell division defect phenotype when PBP2^Y196D^ is the only GT present and SgtB (one of the monofunctional GT in *S. aureus* also called MtgA or MGT) [177] is deleted or inhibited by sub-lethal concentrations of moenomycin. Moreover, the polymerase activity of PBP2^Y196D^ produces shorter glycan chains. The mutated Y196 residue is not critical for catalysis or interaction with the essential pharmacophore of moenomycin. It interacts with ring A and could also interact with the elongating glycan chain; the mutation probably decreases the binding affinity of the polymer for the active site and its premature release, thus yielding glycan chains of reduced length.

### 6.1. GT Inhibitors Based on Moenomycin and Lipid II Substrate

Moenomycin and lipid II substrate have been used as templates for the synthesis of GT inhibitory analogs and have been reviewed recently [178]. In this review, we focus on the most recent studies (Figure 5). Zuegg and colleagues [160] synthesized a disaccharide-based library mimicking the moenomycin EF pharmacophore. The compounds showed good antibacterial activity *in vitro* but had unfavorable physicochemical properties (less active in the presence of 50% serum). Subsequently, on the basis of their data and published results, they designed a library aimed at reducing the molecular mass and the lipophilicity, and used solid-phase combinatorial chemistry to synthesize a library of 500 monosaccharide compounds based on a pyranose scaffold substituted in order to mimic the features of the EF-phosphoglycerate disaccharide moiety of moenomycin. The active compounds (amino-benzimidazole chemotype series) contained lipophilic substituents such as alkyl moieties with a minimum length of 10 carbon atoms or a biaryl one. The two best compounds (**ACL20215** and **ACL20964**, Figure 5) inhibited the activity of the MtgA of *S. aureus* with IC50 values of 17 and 11 µg/mL, respectively, and showed antibacterial activity against resistant and sensitive Gram-positive strains (MIC values against MRSA 4–8 µg/mL) but were inactive on *E. coli*. Interestingly, these compounds are devoid of membrane disruption activity and exhibit low toxicity, good pharmacokinetic properties and *in vivo* efficacy in certain infection model experiments. The **ACL20215** and **ACL20964** compounds were tested at 50 mg·kg^−1^ in a mouse model of septicemia inoculated with *S. aureus* resulting in 100% survival after seven days. Lower dose (4 mg·kg^−1^) administration resulted in only 10% survival. Both compounds cleared 99% of inoculated *S. aureus* from the mouse mammary gland at a moderate dose [160]. In addition, **ACL20215** induced mutation frequencies lower than 2.5 × 10^−10^ on *S. aureus* at 4× MIC. Docking experiments show that the ligand adopts a Y shape with the benzimidazole group located in the phosphoglycerate pocket and the remaining two arms oriented respectively towards the donor (ring E of moenomycin) and the acceptor sites.

Mesleh *et al.* [159] performed computational solvent mapping of a GT to identify druggable pockets in the active site and to help in the design of new compounds. The major hotspot regions were identified in the donor site, overlapping with binding sites of rings EFG of moenomycin A near the essential glutamate and other conserved residues of the active site, confirming that the EFG pharmacophore is the best lead for GT inhibitor design. In addition, a minor hotspot was also found in the acceptor site which would allow an extension of a potential inhibitory compound and bridge the two sites to increase its affinity. In this study the authors also reported the synthesis of 250 disaccharide compounds, some of which demonstrated antibacterial activity against Gram-positive strains (MIC 4–8 µg/mL). However, after further analysis, only two compounds (**ACL19109**, **ACL19110**, Figure 5) were confirmed as specific inhibitors of GT activity. Direct active site binding of the compounds was shown by competition with moenomycin using STD-NMR. Modeling has also emphasized the binding of compounds **ACL19109**, **ACL19110** to the donor site. The biphenyl group overlaps with portions of the lipid tail of moenomycin. The sugars overlap with rings DF of moenomycin A and the two trifluoromethyl benzene groups occupy two hydrophobic pockets near the major hotspot.

The GT is an inverting enzyme that uses an S_N_2 mechanism. Iminosugar C-glycosides based on analogy with the transition state structure of the lipid II substrate during the glycosidic reaction have been explored as GT inhibitors [162]. The synthetic compounds, which contain shorter lipid chains (C15 or C20) than the natural substrate, lack the peptide moiety and have a C-glycosidic linkage to ensure their stability, were able to inhibit the PG synthesis activity of GT *in vitro*. The best compound **1** (Figure 5) has a Ki value of 6.3 µM for the *Clostridium difficile* GT (for moenomycin, Ki = 0.0015 µM). Comparison of different lipid variants (**1**, tetraprenyl *vs.* **21**, triprenyl) (Figure 5) at 100 µM shows that tetrapenyl is a better inhibitor (100% for **1** *vs.* 30% for **21**). Derivatives lacking the lipophilic moiety were inactive. Comparison of two phosphate linkages between the lipid and the iminosugar, phosphonate (**21**, im-C-PP-C15) and elongated phosphate analog (**20**, Figure 5), im-C-O-PP-C15), shows that the increase in the bond length between the lipid and the iminosugar decreases the inhibitory activity (from 30% to 0% at 100 µM) showing that the phosphonate yields a better activity. Uncleavable 1-C-glycoside lipid II analogs, with the minimal required structure consisting of the tetraprenyl (C20) lipid tail and the d-lac-l-Ala peptide, have been investigated as potential GT inhibitors in a study by Lin *et al.* [161]. The comparison of the sugar-phosphate disaccharide-1-C-O-PP-C20 (**1**) (Figure 5) and the sugar-phosphonate disaccharide-1-C-PP-C20 (**2**) (Figure 5) shows that compound **1** has GT inhibitory activity with an IC50 value of 25 µM whereas compound **2** was completely inactive. The monophosphate fragment of **1** lacking the alkyl monophosphate moiety was also inactive. This result contrasts with that obtained with iminosugar compounds where the phosphonate variant was more active [162].

### 6.2. Glycosyltransferases Assays and Screening of Small Molecule Inhibitors

High-throughput screening (HTS) of enzyme inhibitors requires a robust assay and easily available substrates or specific ligands. The available GT assays are rather low- to medium-throughput which prevents large screening campaigns. Because the lipid II substrate is expensive and its synthesis difficult, it is mostly used in low-throughput assays as a radioactive or fluorescent derivative. These assays are useful to confirm the activity of compounds identified by other techniques that are expected to include false positives. Lipid II–based assays have the advantage of detecting lipid II polymerization, and therefore can identify inhibitors that interact with the enzyme or the substrate, and their effects on the donor and acceptor sites, while moenomycin-based assays (see below) only identify ligands binding to the donor site.

A coupled assay consisting of *in situ* synthesis of lipid II and its polymerization by PBPs (GT-TP) was proposed [179]. The assay utilizes *E. coli* cytoplasmic membranes lacking PBP1b which accumulate labeled lipid II from precursors (including radioactive GlcNAc) which are more easily accessible. Subsequently, the membrane fraction containing PBP1b is added. The dual GT-TP activities of PBP1b convert lipid II to cross-linked PG which can be captured with wheat germ agglutinin-coated scintillation proximity beads in the presence of a detergent (Sarkosyl) for quantification. The assay can be performed in microtiter plates for the screening of inhibitors of the GT and TP activities of PBPs.

The most frequently used assay for characterization of the GT is based on purified radioactive lipid II. It is very sensitive and requires a low amount of substrate to quickly analyze the GT activity of an enzyme using TLC [134,180]. For the analysis of both the GT and TP activities the substrate is incubated with a bifunctional PBP, the product is digested by muramidase, and the radioactive muropetides are analyzed by HPLC [155] or, when the unlabeled substrate is used, by LC/MS [45,48]. With the fluorescent substrate (fluorophore bound to the Lys), only the GT activity can be monitored using HPLC [142,148] or LC/MS. Non-digested reaction products formed in the presence of penicillin can be analyzed by SDS-PAGE, which allows the determination of glycan chain length [157]. Omitting penicillin in the reaction mixture enables the identification of cross-linked PG by the TP [44].

A continuous fluorescence assay was developed by Schwartz and colleagues [143] based on dansyl lipid II. The fluorescent probe is attached to the Lys residue of the peptide, thus preventing the cross-linking reaction. During this assay, the polymerase activity of GT is accompanied by a fluorescence decrease when dansyl lipid II is converted to a polymer followed by muramidase hydrolysis. This assay has been further optimized and adapted to microtiter plate format [141,159]. It can be used for the screening of compounds but the presence of fluorescent molecules often interferes with the assay.

**Figure 6:**
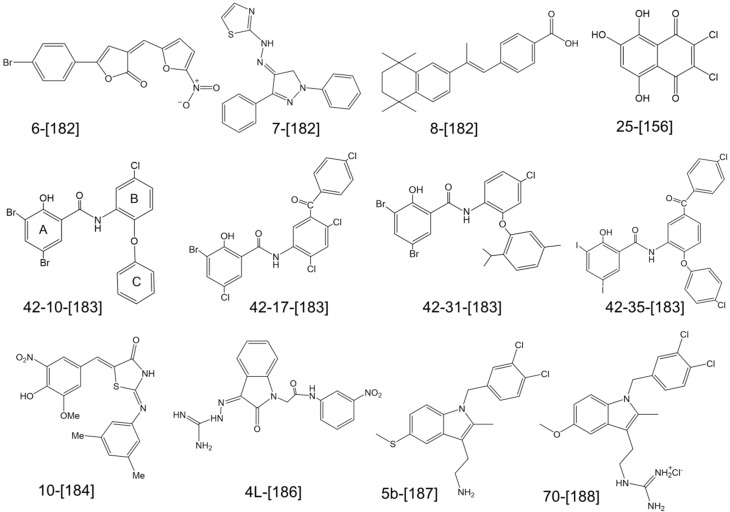
Structures of small molecule inhibitors of glycosyltrasferases. The compounds are numbered as in the original papers and the references are indicated in square brackets.

A Förster resonance energy transfer (FRET-based assay was developed by Huang *et al.* [156] using a C25 lipid II analog labeled with a dimethylamino-azobenzenesulfonyl (dabsyl) quencher at the end of the lipid chain and a coumarin fluorophore attached to the peptide Lys. The substrate alone is not fluorescent, but during the GT reaction, the departure of the pyrophosphate-polyprenol-dabsyl quencher followed by degradation of the polymer by a muramidase allows detection of high-coumarin fluorescence. The FRET assay has been employed to screen a 120,000 compound library. Four known inhibitors have been detected validating the assay; in addition, one new inhibitor (**25**) was identified. Compound **25** (Figure 6) inhibits the GT in a low-micromolar-concentration range (2.3–4.6 µM) and exhibits a MIC value of 16 µg/mL against *S. aureus* but is not active against Gram-negative bacteria.

GT assays based on mass spectrometry analysis have been proposed recently. The first one uses LC/MS to monitor the decrease of the lipid II substrate after the GT reaction [181]. The LC step can be time-consuming and thus may not be suitable for HTS. The second report [159] describes a new GT activity assay developed using the RapidFire High-Throughput MS solid phase extraction system (Agilent Technologies). It is based on the detection of the C55PP product released during the reaction. This assay presents several advantages for HTS. It is based on the lipid II substrate, whose quantity was optimized to reduce the cost, and makes use of rapid solid phase extraction instead of LC separation, which shortens the process and allows fast analysis of a complete 96-well plate in 25 min. Moreover, this assay was found to be more sensitive than LC/MS/MS.

Alternative binding assays that do not rely on lipid II and are based on the displacement of fluorescently labeled moenomycin A (F-Moe) [182,183] or its analog [184] have been used to screen large collections of compounds. A fluorescence anisotropy (FA)-based assay was developed using F-Moe to screen a 57,000 small molecule library (at a concentration of 50 µM) for interaction with full-length *Helicobacter pylori* PBP1a [182]. Eleven hits were initially identified, among which three (**6**, **7**, **8**, Figure 6) were confirmed to bind to the GT (IC50 = 3–34 µM) and exhibit antibacterial activity against *S. aureus* (MIC > 4 µg/mL). Confirmation with lipid II was not reported in this study. In another study [183], cell-based HTS was first performed on a library with two million compound to search for MRSA inhibitors. The positive hits (252 with MIC values against MRSA of 0.03–8 µg/mL) were then tested using the FA assay for competition with F-Moe binding to GT. Sixteen small molecules were identified, but after lipid II polymerization assays, only salicylanilide-based molecules (**42**–**10** and **42**–**17**, Figure 6) were confirmed to inhibit the GT activity *in vitro* (IC50 37 µM). Analogs of these compounds have been synthesized to evaluate their structure-activity relationship. The hydroxyls of ring A and the amide group (Figure 6) were found to be essential for GT inhibition. Salicylanilides with only rings A and B were inactive and the presence of an additional aromatic group on the aniline (ring B) was shown to be important for the inhibition of GT. Compounds (**42**–**31** and **42**–**35**, Figure 6) have good antibacterial activity (1–8 µg/mL) and inhibit the GT competitively with Ki values ranging between 5 and 28 µM depending on the enzymes used.

As moenomycin A binds to the GT with nanomolar affinity, an analog with lower affinity for the GT has been synthesized to allow identification of inhibitors with micromolar affinities [184]. It consisted of a C16-trisaccharide (CEF)-phosphoglycerate substituted with a fluorescein fluorophore on ring C. This probe was used to perform a FA screening assay with a library of 110,000 small molecules against *S. aureus* SgtB. An active compound (**10**, Figure 6) that inhibits PG synthesis *in vitro* was discovered. The IC50 values determined using a lipid II–based GT activity assay were 12–79 µM. The compound also exhibited antibacterial activity with MIC values of 4–16 µg/mL against *S. aureus* and *B. anthracis.*

### 6.3. GT Inhibitor Identified by Structure-Based Virtual Screening

Structure-based virtual screening was employed to search for GT inhibitors. The DrugBank and Life Chemical compounds databases (175,000 compounds) have been filtered based on Lipinski’s rule of five before screening [185]. Nine hits were identified and their potential binding modes verified by visual inspection. However, the enzymatic and antibacterial activities of these compounds were not confirmed experimentally.

High-throughput virtual screening of three million compounds against *S. aureus* PBP2 identified an isatin derivative [186]. Based on this compound, four series of analogs were synthesized to establish structure-activity relationships. The best compound (**4L**, Figure 6) had a MIC value on *S. aureus* of 48 µg/mL and was shown by STD-NMR experiments to compete with moenomycin for binding to the GT.

Using structure-based computational screening, we identified tryptamine derivatives (**5b**, Figure 6) which inhibit the polymerase activity of several GTases with lipid II (IC_50_ 30–60 µM) [187]. The compounds also exhibit antibacterial activity against several Gram-positive bacteria (MIC 4–8 µg/mL) and were confirmed to specifically target cell wall synthesis. While direct binding to the GT could not be established with certainty, we found that compound **5b** interacts with the pyrophosphate motif of lipid II. Analogs of **5b** were recently synthesized and a compound (**70**, Figure 6) with reduced non-specific cytotoxicity but which retains antibacterial and GT inhibition activities was identified [188].

## 7. Conclusions

In the fight against antibiotic resistance, the battlefield is increasingly crowded and each pathogen requires specific attention. To date, ceftaroline and ceftobiprole allow combating Gram-positive bacteria, especially MRSA. However, combating multidrug resistant Gram-negative pathogens constitute the most urgent problem because carbapenems, the present last resort antibiotics, are destroyed by an increasing number of carbapenemases that are now appearing in all classes of β-lactamases. New monocyclic β-lactams bearing a siderophore group are promising. In the same vein, good surprises could come from non β-lactams, especially lactivicins that have already demonstrated high antibacterial potency. Moreover, beyond the development of new antibacterial drugs, synergistic combinations of β-lactam with aminoglycosides, β-lactamase inhibitors, other β-lactams or new products may offer good prospects to extend the lifetime of existing molecules.

New antibiotics are now used with caution but resistances appear rapidly after these drugs are routinely used to treat bacterial infections. The fight against pathogenic bacteria needs recurrent renewal of the antibacterial pipeline. The β-lactam antibiotics remain excellent weapons against bacteria. In this respect, remarkable structural work on TPs and our detailed understanding of the β-lactam-PBP interaction at the atomic level will certainly help in the design of new antibiotics.

The PBP-associated GT domain is a promising target. Although no useful antibiotic has so far been developed against this target, research in this field is intensifying. Two strategies are being pursued to find GT inhibitors, substrate and moenomycin analogs, and small molecules identified by HTS. Structural studies of GT in complex with synthetic analogs or newly discovered small molecules could open original perspectives for the design of effective GT-targeting antibiotics.

Finally, the PBPs are central to the multiprotein complexes elongasome and divisome. These protein-protein interaction networks are essential for peptidoglycan sacculus enlargement and division. In *E. coli*, it has been established that the activation of PBPs by lipoproteins partners is essential for viability. These regulation mechanisms open new perspectives in the search of novel antibacterial. Development of screening assays that take advantage of these processes and report on the perturbation of peptidoglycan synthesis machineries could lead to new antibiotics with novel mechanisms of action.
